# Diagnostic Modalities in the Detection of Cardiac Amyloidosis

**DOI:** 10.3390/jcm13144075

**Published:** 2024-07-12

**Authors:** Syed Bukhari, Zubair Bashir

**Affiliations:** 1 Department of Medicine, Temple University Hospital, Philadelphia, PA 19111, USA; 2 Department of Medicine, Alpert Medical School of Brown University, Providence, RI 02903, USA; zubair_bashir@brown.edu

**Keywords:** cardiac amyloidosis, transthyretin, echocardiography, cardiac magnetic resonance, Tc-pyrophosphate scintigraphy

## Abstract

Cardiac amyloidosis (CA) results mainly from the infiltration of the myocardium by either immunoglobulin light-chain fibrils (AL) or transthyretin fibrils (ATTR), causing restrictive cardiomyopathy and eventually death if untreated. AL derives from monoclonal immunoglobulin light chains produced by plasma cell clones in the bone marrow, while ATTR is the misfolded form of hepatically derived transthyretin (TTR) protein and can be hereditary (ATTRv) or wild-type (ATTRwt). Over the last decade, improvements in diagnostic imaging and better clinical awareness have unleashed a notable presence of CA in the community, especially ATTR in the elderly population. These multimodality imaging modalities include echocardiography, cardiac magnetic resonance, and radionuclide scintigraphy with bone-avid tracers. There has been remarkable progress in the therapeutic landscape as well, and there are disease-modifying therapies available now that can alter the course of the disease and improve survival if initiated at an early stage of the disease. There remains an unmet need for detecting this disease accurately and early so that these patients can benefit the most from newly emerging therapies.

## 1. Introduction

Cardiac amyloidosis (CA) is a rapidly progressing and fatal condition resulting from the interstitial infiltration of misfolded amyloid proteins in the myocardium. The primary proteins involved are either transthyretin (TTR) or light-chain immunoglobulin [1]. TTR, formerly known as prealbumin, is synthesized in the liver and is responsible for transporting retinol and thyroxine in the blood. Its amyloidogenic potential can be hereditary due to point mutations in the TTR gene (ATTRv), or it can occur spontaneously in the elderly (ATTRwt) [1]. ATTRwt predominantly affects males and always involves the heart, while ATTRv shows a range of phenotypes based on the genotype, from neuropathy to cardiomyopathy [2,3]. The V122I mutation, found exclusively in individuals of Afro-Caribbean descent, has a phenotype similar to ATTRwt [4]. Light-chain amyloidosis (AL) is a more aggressive and systemic disease caused by the clonal proliferation of plasma cells in the bone marrow, with a median survival of less than six months from diagnosis if untreated [5]. AL can affect multiple organs, most commonly the kidneys, but cardiac involvement is the major determinant of prognosis.

CA shares many features with other cardiac diseases, including hypertrophic cardiomyopathy and aortic stenosis, and this phenotypic overlap commonly leads to the misdiagnosis or underdiagnosis of CA. CA has been detected in about one-tenth of patients with hypertrophic cardiomyopathy, as well as those with aortic stenosis, and has been diagnosed in ~13% of patients with heart failure with preserved ejection fraction [6,7,8]. The need for easily accessible, cost-effective, and accurate testing that can differentiate CA from its phenocopies has always existed [9].

Advancements in imaging technology have allowed for the non-invasive diagnosis of ATTR, often eliminating the need for tissue confirmation in most cases. Consequently, although the exact prevalence of ATTR remains unknown, a significant prevalence within the community has been revealed in recent years [10]. Simultaneously, new therapeutic drugs for ATTR have emerged, showing notable efficacy in reducing hospitalizations related to heart failure and overall mortality while maintaining an excellent safety profile [11]. These medications are most effective when administered early in the disease progression, underscoring the importance of the timely and early detection of ATTR.

The evolution of multimodality imaging has significantly advanced both the early detection and prognostic assessment of CA. Speckle-tracking echocardiography is often the initial diagnostic tool in clinical practice, identifying non-specific features that raise suspicion for CA and necessitate further diagnostic workup. Nuclear scintigraphy with bone-avid tracers can confirm the diagnosis of ATTR, while histologic confirmation via mass spectrometry is still required to confirm AL. Cardiac magnetic resonance (CMR) is instrumental in differentiating CA from similar conditions, such as hypertrophic cardiomyopathy, sarcoidosis, Anderson–Fabry disease, and cardiac hemochromatosis, and it also provides crucial prognostic information.

This review aims to discuss advancements and the role of multimodality imaging in the diagnostic workup of CA, identify imaging features that could potentially serve as prognostic markers, and highlight some of the knowledge gaps in the field.

## 2. Clinical Recognition of the Disease

The patients often present to the hospital with signs and symptoms of decompensated heart failure, including shortness of breath, paroxysmal nocturnal dyspnea, orthopnea, and lower extremity edema. In rare instances, with advanced disease, hospitalization for cardiogenic shock could be the first presentation. Advanced cases may exhibit ascites, and in rare, severe instances, cardiogenic shock may be the initial presentation [12]. CA patients might experience typical angina, even in the absence of obstructive epicardial coronary artery disease, owing to coronary microvascular dysfunction. The deposition of amyloid fibrils in the interstitium and intramyocardial coronary vessels is thought to increase coronary microvascular resistance, which causes ischemic symptoms [13]. While atrial fibrillation (AF) is the prevailing arrhythmia, bradyarrhythmias and ventricular tachyarrhythmias can serve as the initial presentation in CA [14,15,16]. Elderly patients with ATTRwt may present with low-flow, low-gradient aortic stenosis [17].

Several non-cardiac manifestations of CA are also notable, with musculoskeletal presentations being a distinctive feature of ATTRwt. Carpal tunnel syndrome is caused by amyloid fibril deposition in the flexor retinaculum and tenosynovial tissue within the carpal tunnel. It is the earliest and by far the most prevalent non-cardiac manifestation. About 50% of ATTRwt patients present with carpal tunnel syndrome, and it precedes cardiac involvement by ~7 years on average [18]. In a prospective, cross-sectional study comprising 98 patients who underwent carpal tunnel surgery, Congo red staining of tenosynovial tissue detected amyloid deposits in 10 patients, of whom 2 had cardiac involvement [19]. The CACTUS (Cardiac Amyloidosis Carpal Tunnel Syndrome) study screened patients who were 5 to 15 years post-bilateral carpal tunnel surgery for evidence of CA and found the prevalence to be 5% [20]. Spinal stenosis resulting from amyloid deposition in the ligamentum flavum is exclusively found in ATTRwt. Studies have indicated that about one-third of older adults undergoing lumbar spinal stenosis surgery show amyloid deposition, and its prevalence increases with advancing age [21]. Spontaneous rupture of the distal biceps tendon has been reported in 33% of ATTRwt patients compared to a 2.5% prevalence in non-amyloid heart failure patients, primarily resulting from tendinopathy due to amyloid infiltration [22]. Additionally, ATTRwt patients exhibit a higher prevalence of total hip and knee arthroplasties compared to the general population [23].

AL patients may manifest amyloid involvement at various extra-cardiac sites, except for the brain. Kidney involvement is most commonly seen, often presenting as nephrotic syndrome and proteinuria due to amyloid deposition in the glomeruli. In some cases, amyloid deposition occurs in renal vessels and the tubulointerstitium, leading to renal insufficiency without significant proteinuria [24]. On rare occasions, patients with AL can present with acute kidney injury attributed to intratubular amyloid cast nephropathy. Hepatomegaly in CA patients results from either amyloid infiltration of the liver or congestion secondary to right-sided heart failure. Autonomic nervous system involvement manifests as orthostatic hypotension, gastroparesis, erectile dysfunction, and intestinal dysmotility. Peripheral nervous system involvement causes painful, bilateral, symmetric, distal sensory neuropathy progressing to motor neuropathy. Soft tissue involvement is characterized by macroglossia, an important feature of AL disease.

## 3. Echocardiography

Echocardiography is a non-invasive, accessible, and reproducible imaging technique [25]. Since CA often presents as heart failure, it is typically the first diagnostic tool used for these patients [26]. Echocardiography is vital for measuring key parameters like myocardial wall and valve thickness, myocardial mass, and chamber size, all of which are affected by CA [27,28]. The left ventricular ejection fraction (LVEF) remains preserved until the later stages of CA. As a result, common echocardiographic findings in CA, such as elevated filling pressure, increased wall thickness, and reduced chamber size, resemble those seen in diastolic heart failure [25]. However, certain features specific to CA help differentiate it from other forms of hypertrophic cardiomyopathy (HCM), especially when combined with clinical and demographic data.

Diastolic parameters in CA change significantly over time. In the early stages, patients typically present with a low E-wave, high A-wave velocity, a decreased E/A ratio, and a normal deceleration time. As the disease progresses, the E-wave normalizes, the A-wave diminishes, the E/A ratio increases, and the deceleration time shortens [28]. Additionally, a small S-wave on tissue Doppler is indicative of advanced disease [25]. Tissue Doppler imaging often reveals significantly reduced mitral and tricuspid e′ velocities and a high E/e′ ratio, indicating elevated filling pressures without a marked increase in left ventricular wall thickness, consistent with restrictive myocardial patterns [28,29]. A restrictive pattern on tissue Doppler of the mitral annulus, characterized by e′, a′, and s′ velocities less than 5 cm/s, along with pericardial effusion, strongly suggests CA [24]. Interestingly, tissue Doppler imaging can help identify the different characteristics of the two subtypes; for example, AL-CA typically shows a restrictive pattern earlier in the disease process than ATTR [29].

Left ventricular hypertrophy (LVH) is a key echocardiographic finding in cardiac amyloidosis (CA), presenting symmetrically in AL and asymmetrically in ATTR [30]. The myocardium exhibits a distinctive speckled appearance on echocardiography due to amyloid deposits [31]. The suspicion of CA is heightened when the LV wall thickness exceeds 12 mm and diastolic dysfunction is grade 2 or higher in the absence of aortic valve disease or severe hypertension [32]. Although electrocardiogram (EKG) features such as low voltage in limb leads and poor R-wave progression are not diagnostic of CA, they increase suspicion when considered alongside echocardiographic findings [28,33].

Speckle-tracking echocardiography (STE) reveals a pronounced impairment in left ventricular global longitudinal strain (LVGLS), even in cases where the ejection fraction remains preserved, serving as a significant indicator of a poor prognosis [28]. LVGLS is notably worse in the basal and mid-segments while sparing the apical region, resulting in a distinct apical-sparing pattern [34]. A relative strain ratio exceeding 1 demonstrates high sensitivity and specificity in diagnosing CA [31]. Furthermore, AL typically exhibits more severe LVGLS impairment compared to ATTR-CA at similar levels of wall thickness [35]. An LVEF/GLS ratio surpassing 4.95 emerges as a superior screening tool, offering 75% sensitivity and 66% specificity [36]. Moreover, the integration of three-dimensional STE, incorporating metrics such as global area strain, enhances the sensitivity in detecting CA and evaluating its prognosis [27].

Amyloid deposition within the atrial myocardium results in the thickening of the atrial septum, with measurements surpassing 6 mm demonstrating a diagnostic specificity of 100% for CA [27]. Another common manifestation is biatrial enlargement, driven by heightened filling pressures, serving as an imaging marker for early subclinical changes in ATTR [37].

Additionally, STE shows impairment across all phases of left atrial (LA) strain in CA patients compared to age-matched cohorts without CA. This is likely attributed to the deposition of amyloid fibrils in the atrial myocardium and increased filling pressure from left ventricular diastolic dysfunction [38]. Notably, LA reservoir strain emerges as a promising marker for diastolic dysfunction, with both LA reservoir and contractile strain proving to be superior predictors of elevated left ventricular filling pressure compared to LA volume and conventional Doppler parameters [39]. This underscores the potential of LA strain analysis in the early detection of CA, facilitating the prompt initiation of CA therapies for optimal patient outcomes. Furthermore, in ATTR patients, LA reservoir and contractile strain are lower compared to those with AL or individuals without CA, even in the absence of atrial arrhythmias [40]. Moreover, LA strain can differentiate between CA and HCM, with lower LA strain reported in CA patients compared to those with HCM, despite preserved ejection fraction. Atrial dysfunction observed in CA contributes to a heightened prevalence of thromboembolic events, even among CA patients in sinus rhythm [41]. LA reservoir strain has demonstrated predictive value for AF and thromboembolic events, even in the absence of AF among CA patients [42]. Additionally, LA reservoir strain serves as an independent predictor of cardiovascular-related death and heart failure hospitalization in ATTRwt patients [43].

Moreover, the suspicion of CA is heightened by the unexplained thickening of the right ventricular (RV) wall exceeding 5 mm and accompanied by reduced function [27]. Both speckle-tracking echocardiography (STE) and traditional echocardiography have demonstrated impaired right ventricular free wall longitudinal strain with apical sparing and tricuspid annular plane systolic excursion (TAPSE), respectively [32].

Notably, AL exhibits more significant apical sparing compared to ATTR. An RV apical/(basal + middle) ratio exceeding 0.8 is reported to possess a sensitivity, specificity, and accuracy of 97.8%, 90.0%, and 94.7%, respectively, in distinguishing AL from ATTR [44].

While traditional echocardiography and STE may not serve as the gold standard for diagnosing CA, they play a crucial role in identifying features that aid clinicians in maintaining a high suspicion for CA and progressing to subsequent diagnostic steps in the algorithm for this disease.

## 4. Nuclear Scintigraphy with Bone-Avid Tracers

Originally developed for bone imaging, bone scintigraphy using technetium-99m (99mTc)-labeled radioactive tracers was later discovered to have an affinity for amyloid fibrils in soft tissue. However, it was not until 2005 that the diagnostic potential of 99mTc 2,3-dicarboxypropane-2, 1-diphosphonate (DPD) in identifying ATTR was realized [45]. A seminal European study demonstrated the diagnostic accuracy of 99mTc-DPD, showing 100% differentiation between ATTR and AL, with all ATTR patients (*n* = 15) exhibiting cardiac 99mTc-DPD uptake with Perugini grade ≥ 2, while none in the AL group (*n* = 10) showed tracer uptake and had Perugini grade 0 [45]. Subsequent larger studies, including those using the 99mTc-pyrophosphate (99mTc-PYP) tracer in the US, replicated bone scintigraphy’s high diagnostic performance in diagnosing ATTR [46,47,48]. However, a portion of AL patients also displayed mild tracer uptake (Perugini grade 1), although the exact mechanism of this affinity remains unclear. This affinity is thought to be related to microcalcifications in the amyloid fibrils, with ATTR showing higher microcalcification density compared to AL [49].

**Figure 1:**
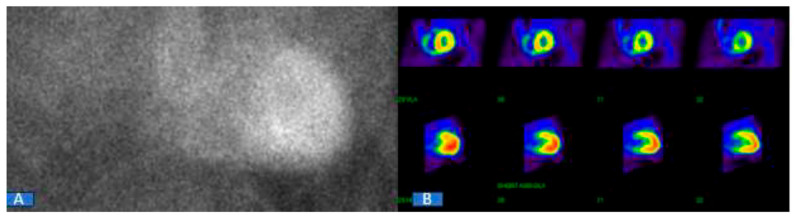
Positive 99mTc-labeled nuclear scintigraphy. (**A**) Visual grade 3 on planar image; (**B**) diffuse myocardial tracer uptake on single-photon emission computed tomography.

**Figure 2:**
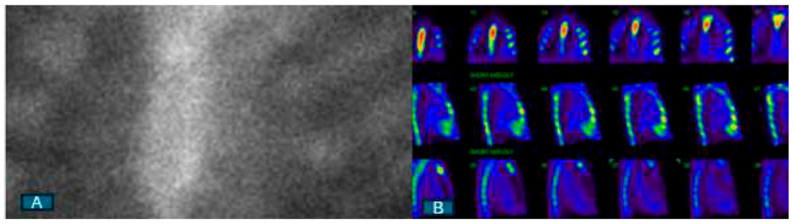
Negative 99mTc-labeled nuclear scintigraphy. (**A**) Visual grade 0 on planar image; (**B**) absence of myocardial tracer uptake on single-photon emission computed tomography.

When CA is suspected based on clinical suspicion and initial imaging findings from echocardiography or CMR, it is imperative to conduct blood and urine tests to assess for evidence of a plasma cell dyscrasia using immunofixation electrophoresis and a free-light-chain assay. Once a plasma cell disorder has been ruled out, consideration should be given to 99mTc-labeled nuclear scintigraphy. The presence of myocardial tracer uptake of grade ≥ 2, without any indication of a plasma cell dyscrasia in blood and urine tests, confirms the diagnosis of ATTR with a specificity and positive predictive value of over 98% (Figure 1). This finding negates the necessity for a biopsy, establishing nuclear scintigraphy as the primary diagnostic imaging modality for patients with suspected ATTR. The absence of myocardial tracer uptake on nuclear scintigraphy essentially rules out ATTR (Figure 2). However, there may be rare instances where a biopsy is still warranted in ATTR patients. For those who display positive scintigraphy with grade ≥ 2 uptake and also present evidence of paraproteinemia in blood and/or urine tests, a biopsy is necessary to explore the potential presence of AL concurrently. It is essential to note that the diagnosis of AL always requires histological confirmation.

Additionally, a biopsy may also be considered for ATTRv patients with specific mutations, like P64L, who exhibit low (grade 1) or absent bone tracer uptake, despite displaying classic clinical, morphological, and functional features on echocardiography and CMR [50]. In such scenarios, tissue diagnosis becomes crucial for definitively confirming cardiac involvement. While the effectiveness of nuclear scintigraphy has been demonstrated in patients referred to amyloid centers with a moderate-to-high pretest probability of CA, its role as a screening tool for ATTR remains undetermined.

## 5. CMR

CMR has diagnostic superiority over echocardiography due to its ability to characterize myocardial tissue. However, it is not the first-line modality because of its cost, the need for specialized expertise, limited widespread use, and restrictions in patients with renal impairment. Often used as an adjunct to echocardiography, CMR can differentiate CA from non-amyloid wall-thickening cardiomyopathies, and it uniquely provides valuable insights into disease prognosis and treatment response.

CMR uses late gadolinium enhancement (LGE) to assess for CA. In CA, amyloid fibril deposition expands the interstitial space between myocardial cells. Gadolinium-based contrast agents accumulate in these enlarged extracellular areas, leading to delayed contrast washout and producing characteristic LGE imaging. Early disease presents a subendocardial pattern, while advanced disease shows a diffuse transmural pattern [51]. LGE can detect CA with high diagnostic sensitivity (85%) and specificity (92%) [52]. Although CMR cannot definitively distinguish between ATTR and AL, LGE is typically more extensive in ATTR, often showing a transmural pattern and involving the right ventricular wall [53]. Additionally, in CA, the time of the inversion scout sequence shows temporal variability in the nulling pattern. An earlier onset of the reverse nulling pattern shows a trend toward a more severe amyloid load and hence more advanced disease [54].

While LGE offers notable advantages, it is not without limitations. Primarily, it functions as a qualitative marker and lacks the capacity to measure amyloid burden accurately. Secondly, the risks of using gadolinium-based contrast outweigh the benefits in individuals with a glomerular filtration rate below 30 mL/min, limiting enhanced imaging. Nonetheless, T1 mapping offers a potential solution to these shortcomings. This technique provides a detailed quantitative analysis of myocardial signals, both before and after contrast administration, at a pixel level. Elevated native (pre-contrast) myocardial T1 levels demonstrate considerable diagnostic accuracy for identifying CA and may even surpass LGE imaging in sensitivity for early disease detection [55]. Native T1 mapping offers valuable predictive values for diagnosing CA, with thresholds of <1036 ms and >1164 ms correlating with 98% negative and positive predictive values, respectively. This could serve as a vital tool in situations where contrast agent use is not feasible. Additionally, native T1 mapping holds the potential for monitoring disease progression in CA. For AL, a poor prognosis is linked with pre-contrast T1 times > 1044 ms, while for ATTR, the threshold is >1077 ms [56,57]. However, native T1 mapping has its limitations. It relies on magnetic field strength and the acquisition technique, and its signal incorporates both extracellular and intracellular factors, hindering its ability to precisely assess amyloid burden.

Combining contrast-enhanced T1 with native T1 allows for the measurement of extracellular volume (ECV), offering a more accurate assessment of amyloid burden [56]. ECV is determined by the ratio of T1 signal changes in the blood and myocardium post-contrast, along with the blood volume of distribution, calculated as 1.0 minus the hematocrit [58]. This marker reflects myocardial tissue remodeling, showing elevation in CA due to infiltrative interstitial expansion. Studies have shown ECV to possess high sensitivity (92%) and specificity (82%) in diagnosing ATTR, also serving as an independent prognostic predictor [59,60]. ECV’s utility extends to evaluating patients with a high pretest probability of CA but negative clinical investigations for cardiac involvement, suggesting its potential as an early disease marker [59].

## 6. Approach to the Diagnosis of CA

The initial suspicion of CA is often raised based on echocardiographic features in the context of a high pretest probability of CA, as described in detail above. Once the clinical suspicion of CA is raised, the next step is to perform nuclear scintigraphy with bone-avid tracers in conjunction with checking serum and urine protein electrophoresis and immunofixation to look for paraproteinemia. A positive 99mTc-PYP scintigraphy result in the absence of paraproteinemia will establish the diagnosis of ATTR, and this will be followed by genetic testing in this scenario to distinguish between ATTRwt and ATTRv. In the case of positive 99mTc-PYP and the concomitant presence of paraproteinemia, histological confirmation with endomyocardial biopsy would be warranted. Alternatively, a bone marrow biopsy can also be performed, which, if negative, will essentially rule out AL. Similarly, if 99mTc-PYP scintigraphy is negative and paraproteinemia is present, a further workup for AL with either an endomyocardial or bone marrow biopsy is warranted. It is important to note that an abdominal fat pad fine-needle aspiration biopsy is another option, but its low sensitivity, particularly in ATTRwt (~15%), as well as the high rate of inadequate specimens, makes it a less reliable test.

Tissue diagnosis may be needed occasionally to detect ATTR when nuclear scintigraphy shows visual grade < 2, urine and serum tests are negative for AL, and clinical, echocardiographic, and/or MRI findings are suggestive of CA. ATTRv associated with the Ser77Tyr and P64L variants has been reported to present with an atypical appearance on nuclear scintigraphy with only grade 1 uptake, despite having classic clinical, morphological, and functional features on echocardiography and CMR [24]. For these patients, tissue diagnosis becomes the definitive method to establish the diagnosis of ATTR.

## 7. Biomarker Profile and CA Staging

Risk stratification, staging, and prognosis currently place great emphasis on blood biomarkers. Natriuretic peptides, including brain natriuretic peptide (BNP) and N-terminal-proBNP, as well as troponin, have been studied extensively for their diagnostic and prognostic value in CA. While elevated BNP and NT-proBNP are universally present in CA, a chronically elevated level of serum troponin is not uncommon in CA and predicts a poor prognosis [61].

Several staging systems using biomarkers have been proposed in CA for the assessment of the severity of the disease. Two staging systems exist for ATTRwt. The first one used thresholds of troponin T (0.05 ng/mL) and NT-proBNP (3000 pg/mL). The 4-year overall survival was estimated to be 57%, 42%, and 18% for stage I (both values below cutoff), stage II (one value above cutoff), and stage III (both above cutoff), respectively [62,63]. A subsequent study that examined both ATTRwt and ATTRv substituted troponin with an estimated glomerular filtration rate of 45 mL/min as the threshold. Median survival was 69, 47, and 24 months in stages I, II, and III, respectively [64].

For AL, the Mayo staging system initially proposed the utilization of both troponin (troponin T < 0.035 µg/L or troponin I < 0.1 µg/L) and NT-proBNP (<322 ng/L) and defined stages I, II, and III based on whether neither, one, or both of these markers are above the normal limit [65,66]. Stages I, II, and III predicted a median survival of 27.2, 11.1, and 4.1 months, respectively. A subsequent staging system comprising the difference between involved and uninvolved free light chains (FLC-diff), troponin T, and NT-proBNP defined stages I, II, III, and IV based on whether none, one, two, or all three factors were above the normal limit and correlated with a median overall survival of 94, 40, 14, and 6 months, respectively [67].

## 8. Knowledge Gaps and Directions for Future

The emergence of sophisticated imaging techniques has enabled the non-invasive diagnosis of CA. There still remain knowledge gaps that need to be addressed. While the sensitivity and specificity of scintigraphy to diagnose ATTR are high when there is grade ≥ 2 uptake in the absence of paraproteinemia, the sensitivity decreases sharply when paraproteinemia is excluded from the algorithm. It is also unsuitable for the diagnosis of AL and for rare forms of CA. In addition, there are certain ATTR mutations that cannot be detected using nuclear scintigraphy, posing another challenge to this rapidly evolving field of multimodality imaging.

There is a need for a risk calculator, using imaging markers, to accurately assess the disease burden, progression, and prognosis. Most of the current tools for staging are based on laboratory markers, and hence, with the advancements in multimodality imaging, there remains a need for imaging-based risk assessment tools. The capability of serial imaging at multiple time points to determine treatment response and to identify non-responders has not yet been assessed.

## 9. Conclusions

The emergence of sophisticated imaging modalities has revolutionized the field of CA and has helped in the timely detection of the disease. Echocardiography is often the first test that raises suspicion for CA. CMR can easily distinguish CA from other etiologies of hypertrophic cardiomyopathy and can also help to monitor disease progression as well as predict prognosis. Nuclear scintigraphy with bone tracers establishes the diagnosis of ATTR in the absence of paraproteinemia, circumventing the need for endomyocardial biopsy in the majority of cases. AL cannot be confirmed by imaging and requires a biopsy.

## References

[1] Merlini G., Bellotti V. (2003). Molecular Mechanisms of Amyloidosis. N. Engl. J. Med..

[2] Masri A., Bukhari S., Eisele Y.S., Soman P. (2020). Molecular Imaging of Cardiac Amyloidosis. J. Nucl. Med..

[3] Pilebro B., Suhr O.B., Näslund U., Westermark P., Lindqvist P., Sundström T. (2016). (99m)Tc-DPD uptake reflects amyloid fibril composition in hereditary transthyretin amyloidosis. Ups. J. Med. Sci..

[4] Buxbaum J., Jacobson D.R., Tagoe C., Alexander A., Kitzman D.W., Greenberg B., Thaneemit-Chen S., Lavori P. (2006). Transthyretin V122I in African Americans with congestive heart failure. J. Am. Coll. Cardiol..

[5] Kyle R.A., Linos A., Beard C.M., Linke R.P., Gertz M.A., O’Fallon W.M., Kurland L.T. (1992). Incidence and natural history of primary systemic amyloidosis in Olmsted County, Minnesota, 1950 through 1989. Blood.

[6] González-López E., Gallego-Delgado M., Guzzo-Merello G., de Haro-Del Moral F.J., Cobo-Marcos M., Robles C., Bornstein B., Salas C., Lara-Pezzi E., Alonso-Pulpon L. (2015). Wild-type transthyretin amyloidosis as a cause of heart failure with preserved ejection fraction. Eur. Heart J..

[7] Castaño A., Narotsky D.L., Hamid N., Khalique O.K., Morgenstern R., DeLuca A., Rubin J., Chiuzan C., Nazif T., Vahl T. (2017). Unveiling transthyretin cardiac amyloidosis and its predictors among elderly patients with severe aortic stenosis undergoing transcatheter aortic valve replacement. Eur. Heart J..

[8] Maurizi N., Rella V., Fumagalli C., Salerno S., Castelletti S., Dagradi F., Torchio M., Marceca A., Meda M., Gasparini M. (2020). Prevalence of cardiac amyloidosis among adult patients referred to tertiary centres with an initial diagnosis of hypertrophic cardiomyopathy. Int. J. Cardiol..

[9] Bukhari S., Khan S.Z., Ghoweba M., Khan B., Bashir Z. (2024). Arrhythmias and Device Therapies in Cardiac Amyloidosis. J. Clin. Med..

[10] Ravichandran S., Lachmann H.J., Wechalekar A.D. (2020). Epidemiologic and Survival Trends in Amyloidosis, 1987–2019. N. Engl. J. Med..

[11] Maurer M.S., Schwartz J.H., Gundapaneni B., Elliott P.M., Merlini G., Waddington-Cruz M., Kristen A.V., Grogan M., Witteles R., Damy T. (2018). Tafamidis Treatment for Patients with Transthyretin Amyloid Cardiomyopathy. N. Engl. J. Med..

[12] Oye M., Dhruva P., Kandah F., Oye M., Missov E. (2021). Cardiac amyloid presenting as cardiogenic shock: Case series. Eur. Heart J. Case Rep..

[13] Dorbala S., Vangala D., Bruyere J., Quarta C., Kruger J., Padera R., Foster C., Hanley M., Di Carli M.F., Falk R. (2014). Coronary microvascular dysfunction is related to abnormalities in myocardial structure and function in cardiac amyloidosis. JACC Heart Fail..

[14] Bukhari S., Kasi A., Khan B. (2023). Bradyarrhythmias in Cardiac Amyloidosis and Role of Pacemaker. Curr. Probl. Cardiol..

[15] Bukhari S., Khan B. (2023). Prevalence of ventricular arrhythmias and role of implantable cardioverter-defibrillator in cardiac amyloidosis. J. Cardiol..

[16] Bukhari S., Barakat A.F., Eisele Y.S., Nieves R., Jain S., Saba S., Follansbee W.P., Brownell A., Soman P. (2021). Prevalence of Atrial Fibrillation and Thromboembolic Risk in Wild-Type Transthyretin Amyloid Cardiomyopathy. Circulation.

[17] Nitsche C., Scully P.R., Patel K.P., Kammerlander A.A., Koschutnik M., Dona C., Wollenweber T., Ahmed N., Thornton G.D., Kelion A.D. (2021). Prevalence and Outcomes of Concomitant Aortic Stenosis and Cardiac Amyloidosis. J. Am. Coll. Cardiol..

[18] Milandri A., Farioli A., Gagliardi C., Longhi S., Salvi F., Curti S., Foffi S., Caponetti A.G., Lorenzini M., Ferlini A. (2020). Carpal tunnel syndrome in cardiac amyloidosis: Implications for early diagnosis and prognostic role across the spectrum of aetiologies. Eur. J. Heart Fail..

[19] Sperry B.W., Reyes B.A., Ikram A., Donnelly J.P., Phelan D., Jaber W.A., Shapiro D., Evans P.J., Maschke S., Kilpatrick S.E. (2018). Tenosynovial and Cardiac Amyloidosis in Patients Undergoing Carpal Tunnel Release. J. Am. Coll. Cardiol..

[20] Westin O., Fosbøl E.L., Maurer M.S., Leicht B.P., Hasbak P., Mylin A.K., Rørvig S., Lindkær T.H., Johannesen H.H., Gustafsson F. (2022). Screening for Cardiac Amyloidosis 5 to 15 Years After Surgery for Bilateral Carpal Tunnel Syndrome. J. Am. Coll. Cardiol..

[21] Maurer M.S., Smiley D., Simsolo E., Remotti F., Bustamante A., Teruya S., Helmke S., Einstein A.J., Lehman R., Giles J.T. (2022). Analysis of lumbar spine stenosis specimens for identification of amyloid. J. Am. Geriatr. Soc..

[22] Geller H.I., Singh A., Alexander K.M., Mirto T.M., Falk R.H. (2017). Association Between Ruptured Distal Biceps Tendon and Wild-Type Transthyretin Cardiac Amyloidosis. J. Am. Med. Assoc..

[23] Rubin J., Alvarez J., Teruya S., Castano A., Lehman R.A., Weidenbaum M., Geller J.A., Helmke S., Maurer M.S. (2017). Hip and knee arthroplasty are common among patients with transthyretin cardiac amyloidosis, occurring years before cardiac amyloid diagnosis: Can we identify affected patients earlier?. Amyloid.

[24] Bukhari S. (2023). Cardiac amyloidosis: State-of-the-art review. J. Geriatr. Cardiol..

[25] Cuddy S.A.M., Chetrit M., Jankowski M., Desai M., Falk R.H., Weiner R.B., Klein A.L., Phelan D., Grogan M. (2022). Practical Points for Echocardiography in Cardiac Amyloidosis. J. Am. Soc. Echocardiogr..

[26] McDonagh T.A., Metra M., Adamo M., Gardner R.S., Baumbach A., Böhm M., Burri H., Butler J., Čelutkienė J., Chioncel O. (2021). 2021 ESC Guidelines for the diagnosis and treatment of acute and chronic heart failure. Eur. Heart J..

[27] Liang S., Liu Z., Li Q., He W., Huang H. (2023). Advance of echocardiography in cardiac amyloidosis. Heart Fail. Rev..

[28] Dorbala S., Cuddy S., Falk R.H. (2020). How to Image Cardiac Amyloidosis: A Practical Approach. JACC Cardiovasc. Imaging.

[29] Rapezzi C., Aimo A., Barison A., Emdin M., Porcari A., Linhart A., Keren A., Merlo M., Sinagra G. (2022). Restrictive cardiomyopathy: Definition and diagnosis. Eur. Heart J..

[30] Martinez-Naharro A., Baksi A.J., Hawkins P.N., Fontana M. (2020). Diagnostic imaging of cardiac amyloidosis. Nat. Rev. Cardiol..

[31] Bashir Z., Chen E.W., Tori K., Ghosalkar D., Aurigemma G.P., Dickey J.B., Haines P. (2023). Insight into different phenotypic presentations of heart failure with preserved ejection fraction. Prog. Cardiovasc. Dis..

[32] Dorbala S., Ando Y., Bokhari S., Dispenzieri A., Falk R.H., Ferrari V.A., Fontana M., Gheysens O., Gillmore J.D., Glaudemans A.W.J.M. (2021). ASNC/AHA/ASE/EANM/HFSA/ISA/SCMR/SNMMI Expert Consensus Recommendations for Multimodality Imaging in Cardiac Amyloidosis: Part 2 of 2-Diagnostic Criteria and Appropriate Utilization. Circ. Cardiovasc. Imaging.

[33] Ng P.L.F., Lim Y.C., Evangelista L.K.M., Wong R.C.C., Chai P., Sia C.H., Loi H.Y., Yeo T.C., Lin W. (2022). Utility and pitfalls of the electrocardiogram in the evaluation of cardiac amyloidosis. Ann. Noninvasive Electrocardiol..

[34] Bravo P.E., Fujikura K., Kijewski M.F., Jerosch-Herold M., Jacob S., El-Sady M.S., Sticka W., Dubey S., Belanger A., Park M.A. (2019). Relative Apical Sparing of Myocardial Longitudinal Strain Is Explained by Regional Differences in Total Amyloid Mass Rather Than the Proportion of Amyloid Deposits. JACC Cardiovasc. Imaging.

[35] Quarta C.C., Solomon S.D., Uraizee I., Kruger J., Longhi S., Ferlito M., Gagliardi C., Milandri A., Rapezzi C., Falk R.H. (2014). Left ventricular structure and function in transthyretin-related versus light-chain cardiac amyloidosis. Circulation.

[36] Kyrouac D., Schiffer W., Lennep B., Fergestrom N., Zhang K.W., Gorcsan J., Lenihan D.J., Mitchell J.D. (2022). Echocardiographic and clinical predictors of cardiac amyloidosis: Limitations of apical sparing. ESC Heart Fail..

[37] Minamisawa M., Inciardi R.M., Claggett B., Cuddy S.A.M., Quarta C.C., Shah A.M., Dorbala S., Falk R.H., Matsushita K., Kitzman D.W. (2021). Left atrial structure and function of the amyloidogenic V122I transthyretin variant in elderly African Americans. Eur. J. Heart Fail..

[38] Bashir Z., Younus A., Dhillon S., Kasi A., Bukhari S. (2024). EXPRESS: Epidemiology, Diagnosis and Management of Cardiac Amyloidosis. J. Investig. Med..

[39] Inoue K., Khan F.H., Remme E.W., Ohte N., García-Izquierdo E., Chetrit M., Moñivas-Palomero V., Mingo-Santos S., Andersen Ø.S., Gude E. (2021). Determinants of left atrial reservoir and pump strain and use of atrial strain for evaluation of left ventricular filling pressure. Eur. Heart J. Cardiovasc. Imaging.

[40] Aimo A., Fabiani I., Giannoni A., Mandoli G.E., Pastore M.C., Vergaro G., Spini V., Chubuchny V., Pasanisi E.M., Petersen C. (2022). Multi-chamber speckle tracking imaging and diagnostic value of left atrial strain in cardiac amyloidosis. Eur. Heart J. Cardiovasc. Imaging.

[41] Bukhari S., Khan S.Z., Bashir Z. (2023). Atrial Fibrillation, Thromboembolic Risk, and Anticoagulation in Cardiac Amyloidosis: A Review. J. Card. Fail..

[42] Akintoye E., Majid M., Klein A.L., Hanna M. (2023). Prognostic Utility of Left Atrial Strain to Predict Thrombotic Events and Mortality in Amyloid Cardiomyopathy. JACC Cardiovasc. Imaging.

[43] Bukhari S., Oliveros E., Parekh H., Farmakis D. (2023). Epidemiology, Mechanisms, and Management of Atrial Fibrillation in Cardiac Amyloidosis. Curr. Probl. Cardiol..

[44] Moñivas Palomero V., Durante-Lopez A., Sanabria M.T., Cubero J.S., González-Mirelis J., Lopez-Ibor J.V., Navarro Rico S.M., Krsnik I., Dominguez F., Mingo A.M. (2019). Role of Right Ventricular Strain Measured by Two-Dimensional Echocardiography in the Diagnosis of Cardiac Amyloidosis. J. Am. Soc. Echocardiogr..

[45] Perugini E., Guidalotti P.L., Salvi F., Cooke R.M., Pettinato C., Riva L., Leone O., Farsad M., Ciliberti P., Bacchi-Reggiani L. (2005). Noninvasive etiologic diagnosis of cardiac amyloidosis using 99mTc-3,3-diphosphono-1,2-propanodicarboxylic acid scintigraphy. J. Am. Coll. Cardiol..

[46] Bokhari S., Castaño A., Pozniakoff T., Deslisle S., Latif F., Maurer M.S. (2013). (99m)Tc-pyrophosphate scintigraphy for differentiating light-chain cardiac amyloidosis from the transthyretin-related familial and senile cardiac amyloidoses. Circ. Cardiovasc. Imaging.

[47] Gillmore J.D., Maurer M.S., Falk R.H., Merlini G., Damy T., Dispenzieri A., Wechalekar A.D., Berk J.L., Quarta C.C., Grogan M. (2016). Nonbiopsy Diagnosis of Cardiac Transthyretin Amyloidosis. Circulation.

[48] Masri A., Bukhari S., Ahmad S., Nieves R., Eisele Y.S., Follansbee W., Brownell A., Wong T.C., Schelbert E., Soman P. (2020). Efficient 1-Hour Technetium-99 m Pyrophosphate Imaging Protocol for the Diagnosis of Transthyretin Cardiac Amyloidosis. Circ. Cardiovasc. Imaging.

[49] Stats M.A., Stone J.R. (2016). Varying levels of small microcalcifications and macrophages in ATTR and AL cardiac amyloidosis: Implications for utilizing nuclear medicine studies to subtype amyloidosis. Cardiovasc. Pathol..

[50] Musumeci M.B., Cappelli F., Russo D., Tini G., Canepa M., Milandri A., Bonfiglioli R., Di Bella G., My F., Luigetti M. (2020). Low Sensitivity of Bone Scintigraphy in Detecting Phe64Leu Mutation-Related Transthyretin Cardiac Amyloidosis. JACC Cardiovasc. Imaging.

[51] Maceira A.M., Joshi J., Prasad S.K., Moon J.C., Perugini E., Harding I., Sheppard M.N., Poole-Wilson P.A., Hawkins P.N., Pennell D.J. (2005). Cardiovascular Magnetic Resonance in Cardiac Amyloidosis. Circulation.

[52] Zhao L., Tian Z., Fang Q. (2016). Diagnostic accuracy of cardiovascular magnetic resonance for patients with suspected cardiac amyloidosis: A systematic review and meta-analysis. BMC Cardiovasc. Disord..

[53] Dungu J.N., Valencia O., Pinney J.H., Gibbs S.D., Rowczenio D., Gilbertson J.A., Lachmann H.J., Wechalekar A., Gillmore J.D., Whelan C.J. (2014). CMR-Based Differentiation of AL and ATTR Cardiac Amyloidosis. JACC Cardiovasc. Imaging.

[54] Mahalingam H., Chacko B.R., Irodi A., Joseph E., Vimala L.R., Thomson V.S. (2018). Myocardial nulling pattern in cardiac amyloidosis on time of inversion scout magnetic resonance imaging sequence—A new observation of temporal variability. Indian J. Radiol. Imaging.

[55] Karamitsos T.D., Piechnik S.K., Banypersad S.M., Fontana M., Ntusi N.B., Ferreira V.M., Whelan C.J., Myerson S.G., Robson M.D., Hawkins P.N. (2013). Noncontrast T1 Mapping for the Diagnosis of Cardiac Amyloidosis. JACC Cardiovasc. Imaging.

[56] Banypersad S.M., Fontana M., Maestrini V., Sado D.M., Captur G., Petrie A., Piechnik S.K., Whelan C.J., Herrey A.S., Gillmore J.D. (2015). T1 mapping and survival in systemic light-chain amyloidosis. Eur. Heart J..

[57] Martinez-Naharro A., Kotecha T., Norrington K., Boldrini M., Rezk T., Quarta C., Treibel T.A., Whelan C.J., Knight D.S., Kellman P. (2019). Native T1 and Extracellular Volume in Transthyretin Amyloidosis. JACC Cardiovasc. Imaging.

[58] Banypersad S.M., Sado D.M., Flett A.S., Gibbs S.D., Pinney J.H., Maestrini V., Cox A.T., Fontana M., Whelan C.J., Wechalekar A.D. (2013). Quantification of myocardial extracellular volume fraction in systemic AL amyloidosis: An equilibrium contrast cardiovascular magnetic resonance study. Circ. Cardiovasc. Imaging.

[59] Nieves R.A., Bukhari S., Harinstein M.E. (2021). Adding value to myocardial perfusion scintigraphy: A prediction tool to predict adverse cardiac outcomes and risk stratify. J. Nucl. Cardiol..

[60] Olausson E., Wertz J., Fridman Y., Bering P., Maanja M., Niklasson L., Wong T.C., Fukui M., Cavalcante J.L., Cater G. (2023). Diffuse myocardial fibrosis associates with incident ventricular arrhythmia in implantable cardioverter defibrillator recipients. medRxiv.

[61] Takashio S., Yamamuro M., Izumiya Y., Hirakawa K., Marume K., Yamamoto M., Ueda M., Yamashita T., Ishibashi-Ueda H., Yasuda S. (2018). Diagnostic utility of cardiac troponin T level in patients with cardiac amyloidosis. ESC Heart Fail..

[62] Grogan M., Scott C.G., Kyle R.A., Zeldenrust S.R., Gertz M.A., Lin G., Klarich K.W., Miller W.L., Maleszewski J.J., Dispenzieri A. (2016). Natural History of Wild-Type Transthyretin Cardiac Amyloidosis and Risk Stratification Using a Novel Staging System. J. Am. Coll. Cardiol..

[63] Elgendy I.Y., Bukhari S., Barakat A.F., Pepine C.J., Lindley K.J., Miller E.C. (2021). American College of Cardiology Cardiovascular Disease in Women Committee. Maternal Stroke: A Call for Action. Circulation.

[64] Gillmore J.D., Damy T., Fontana M., Hutchinson M., Lachmann H.J., Martinez-Naharro A., Quarta C.C., Rezk T., Whelan C.J., Gonzalez-Lopez E. (2018). A new staging system for cardiac transthyretin amyloidosis. Eur. Heart J..

[65] Dispenzieri A., Gertz M.A., Kyle R.A., Lacy M.Q., Burritt M.F., Therneau T.M., Greipp P.R., Witzig T.E., Lust J.A., Rajkumar S.V. (2004). Serum cardiac troponins and N-terminal pro-brain natriuretic peptide: A staging system for primary systemic amyloidosis. J. Clin. Oncol..

[66] Bukhari S., Yaghi S., Bashir Z. (2023). Stroke in Young Adults. J. Clin. Med..

[67] Kumar S., Dispenzieri A., Lacy M.Q., Hayman S.R., Buadi F.K., Colby C., Laumann K., Zeldenrust S.R., Leung N., Dingli D. (2012). Revised prognostic staging system for light chain amyloidosis incorporating cardiac biomarkers and serum free light chain measurements. J. Clin. Oncol..

